# Both left upper lobectomy and left pneumonectomy are risk factors for postoperative stroke

**DOI:** 10.1038/s41598-019-46989-w

**Published:** 2019-07-18

**Authors:** Nanchang Xie, Xianghe Meng, Chuanjie Wu, Yajun Lian, Cui Wang, Mengyan Yu, Yingjiao Li, Yali Wang

**Affiliations:** 1 Department of Neurology, The First Affiliated Hospital of Zhengzhou University, Zhengzhou, 450052 China; 2 Department of Neurology, Xuanwu Hospital Capital Medical University, Beijing, 100000 China; 3 Department of Clinical Laboratory, The First Affiliated Hospital of Zhengzhou University, Zhengzhou, 450052 China

**Keywords:** Risk factors, Stroke

## Abstract

Retrospective studies have found that left upper lobectomy (LUL) may be a new risk factor for stroke, and the potential mechanism is pulmonary vein thrombosis, which more likely develops in the left superior pulmonary vein (LSPV) stump. The LSPV remaining after left pneumonectomy is similar to that remaining after LUL. However, the association between left pneumonectomy, LUL, and postoperative stroke remains unclear. Thus, we sought to analyze whether both LUL and left pneumonectomy are risk factors for postoperative stroke. We prospectively included consecutive patients who underwent resection between November 2016 and March 2018 at our institution with 6 months of follow-up. Baseline demographic and clinical data were taken. A logistic regression model was used to determine independent predictors of postoperative stroke. In our study, 756 patients who underwent an isolated pulmonary lobectomy procedure were screened; of these, 637 patients who completed the 6-month follow-up were included in the analysis. Multivariable logistic regression analysis adjusted for common risk factors showed that the LUL and left pneumonectomy were independent predictors of stroke (odds ratio, 18.12; 95% confidence interval, 2.12–155.24; P = 0.008). Moreover, diabetes mellitus also was a predictor of postoperative stroke. In conclusion, both LUL and left pneumonectomy are significant risk factors for postoperative stroke.

## Introduction

Stroke is one of the most feared complications of surgery, which occurs in 0.08–0.7% and 0.6% of general and thoracic surgery patients, respectively^1–3^. The mortality is approximately 15% after the first stroke in the general population, whereas the acute mortality following a perioperative stroke is approximately 26%^4^. Long-term neurological disabilities following postoperative stroke result in a substantial social and financial burden to the family and the community. According to large-scale database studies, advanced age, ischemic heart disease, history of cerebrovascular disease, atrial fibrillation, and congestive heart failure are the common risk factors for perioperative stroke^5^.

Major cardiovascular surgery around a relatively high incidence of perioperative stroke^6,7^. Recent studies found that left upper lobectomy (LUL) may be a new risk factor for stroke in cancer patients^8,9^, and the potential mechanism is pulmonary vein thrombosis, which more likely forms in the left superior pulmonary vein (LSPV) stump^9,10^. Interestingly, a giant thrombus in the LSPV after left pneumonectomy has been reported^11^. It is known that the LSPV remaining after left pneumonectomy is the same as that after LUL. Thus, we speculate that left pneumonectomy may also be a risk factor for postoperative stroke. However, there is only one case report that mentions the relationship between left pneumonectomy and stroke^12^. In addition, these studies are retrospective in nature and are only performed in patients with lung cancer, and whether this conclusion is applicable to patients other than lung cancer remains relatively unknown.

In this prospective cohort study, we sought to determine whether both LUL and left pneumonectomy are risk factors for postoperative stroke in patients with lung cancer, inflammatory pseudotumor, bronchiectasis, and other pathological diseases who underwent lobectomy.

## Methods

### Study design and patient selection

This prospective cohort study was designed to provide data on postoperative stroke risk factors of patients who underwent pulmonary lobectomy in our institution. We used the STROBE checklist. Eligible patients were enrolled consecutively from November 2016 to March 2018 at the First Affiliated Hospital of Zhengzhou University. The follow-up time of this study was 6 months after surgery. LUL and left pneumonectomy were compared with other operative procedures in these patients.

The study protocol was approved by the institutional review committee of the First Affiliated Hospital of Zhengzhou University (project identification code: 2018-LW-043), and informed consent was obtained from the patients prior to study participation. The study was performed according to the institution’s ethical guideline and the principles of the Helsinki Declaration.

We prospectively and consecutively selected patients. Inclusion criteria were as follows: (1) age between 18 and 80 years, (2) pulmonary lobectomy, bilobectomy, or pneumonectomy was planned at our hospital, and (3) signed the informed consent form. Exclusion criteria were as follows: (1) life expectancy was less than 6 months, (2) a patient who had a surgical history of lobectomy, bilobectomy, or pneumonectomy, (3) formerly known abnormalities of any coagulation factor, (4) use of unfractionated heparin, low molecular weight heparin (LMWH), and any form of anticoagulant and antiplatelet drug in the past 30 days, (5) history of previous cardiovascular surgery (coronary artery bypass surgery or percutaneous coronary intervention), (6) history of previous stroke, (7) pregnant, lactating, or potentially pregnant, (8) uncontrollable infectious disease, autoimmune disease, or other severe comorbidities, (9) psychiatric disease, and (10) inaccessible to follow-up.

### Surgery and pathology

All patients presented to our hospital for a routine preoperative check-up and underwent computed tomography (CT) of the chest and abdomen and magnetic resonance imaging (MRI) of the brain. For all patients (except for large tumor size, lymph node metastases, bronchial invasion, chest wall invasion or pulmonary vessel invasion on preoperative images), video-assisted thoracoscopic surgery (VATS) with a 7-cm small thoracotomy, 3-cm window, and three ports were executed. For the other patients, a thoracotomy of 13 to 20 cm was performed. All patients underwent a standard procedure for anatomical pulmonary surgery. The pulmonary vein, pulmonary artery, and bronchus were all divided by a linear stapler. Tumor pathological typing was according to the 2015 World Health Organization Classification of Lung Tumors^13^.

### Postoperative venous thrombosis prevention

For all patients, hemagglutination test was performed within 24 hours after surgery and followed with venous thrombosis preventive measures.

According to the American College of Chest Physicians Evidence-Based Clinical Practice Guidelines (8th Edition) for the prevention of venous thromboembolism^14^, we selected the following postoperative venous thrombosis preventive procedures: (1) routine thromboprophylaxis with LMWH [international normalized ratio (INR) target, 2.5; range, 2.0 to 3.0] was administered for patients who underwent major thoracic surgery; and (2) mechanical thromboprophylaxis with properly fitted graduated compression stockings (GCS) was utilized for thoracic surgery patients who had a high risk of bleeding.

### Outcome of interest

The outcome of interest was incident ischemic stroke during the 6-month follow-up period. Ischemic stroke was defined as a sudden onset of focal neurological deficit that lasts for more than 24 hours and is not due to non-ischemic causes; if the neurological deficit lasts less than 24 hours, evidence of acute cerebral infarction must appear in the neuroimaging examination^15^.

Suspected ischemic strokes, which were assessed by trained and research neurologists, were reported via telephone follow-up with participants every month within 6 months after surgery. Medical records were requested for ischemic stroke events and reviewed by two experienced neurologists, both blinded to the study design and baseline characteristics, who also validated potential strokes. Differences were resolved by discussion and a consensus was reached between the two neurologists. If consensus couldn’t be reached, the third reviewer was involved in resolving any differences. All patients who might have an incident stroke during follow-up underwent head magnetic resonance imaging (MRI) or computed tomography (CT).

### Statistical analysis

SPSS version 21.0 (SPSS Inc., Chicago, IL, USA) was used for all statistical analyses. Frequencies were tabulated for each patient’s risk factor. The results are expressed as percentages for categorical variables and as mean (standard deviation, SD) for continuous variables. A univariable analysis was performed using Chi-squared or Fisher’s exact tests for categorical variables or continuous variables. Student’s t test or Mann-Whitney U test was used to compare patients with and without postoperative stroke. All variables showing significance in the nominal two-tailed test (P < 0.10) and traditional cerebral infarction risk factors [age, sex, smoking index (smoking index = the number of cigarettes smoked per day (CPD) × years of tobacco use^16^), drinking history, hypertension, diabetes mellitus, hyperlipidemia, arrhythmia, coronary heart disease, hypercholesterolemia, type of procedure, operative approach (thoracotomy/VATS)] were entered into a binary logistic regression model using an enter selection method. We report the unadjusted and adjusted values, with 95% confidence intervals (CIs) to indicate statistical accuracy. A P < 0.05 (two-sided) was considered statistically significant.

## Results

### Study participants and baseline characteristics

**Figure 1:**
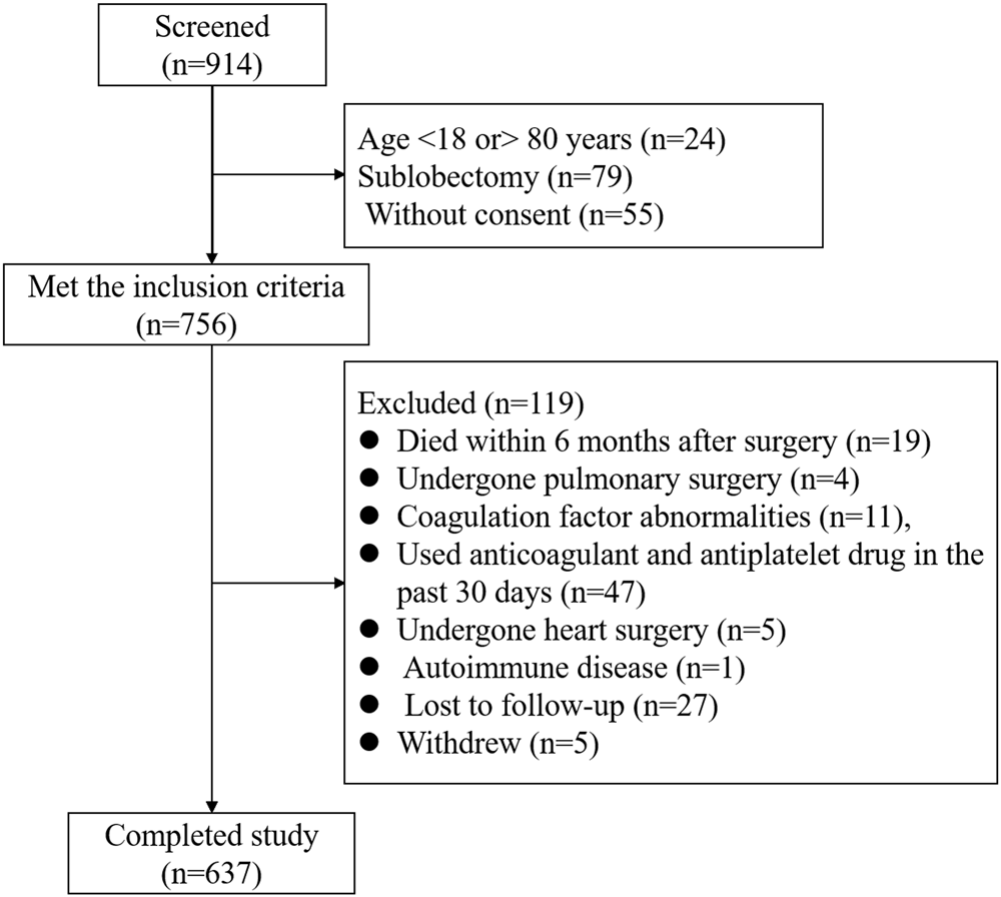
Enrollment and Outcome. Of 914 patients screened, 637 patients were analyzed.

From November 2016 to March 2018, a total of 914 patients were screened consecutively; of these, 756 patients met the inclusion criteria, whereas 119 patients were excluded (19 patients died within 6 months after surgery, 4 had undergone pulmonary surgery, 11 had coagulation factor abnormalities, 47 used anticoagulant and antiplatelet drug in the past 30 days, 5 had undergone heart surgery, 1 had an autoimmune disease, 27 was lost to follow-up, and 5 withdrew). In this study, 637 patients were enrolled for analysis (Fig. 1). The median age of patients included in this study was 60 [interquartile range (IQR), 53–66) years, and 61.07% of the patients were male. The baseline characteristics of the 637 patients who had an isolated pulmonary lobectomy procedure and completed the 6-month follow-up were described in Table 1.

**Table 1:** Results of univariate analysis of stroke.

Variable	Total n = 637	Stroke n = 10	No stroke n = 627	P-value
Age (years)	59.1 ± 9.8	61.6 ± 7.4	59.1 ± 9.9	0.424
Sex n(%)				0.098
Male	389 (61.1)	9 (2.3)	380 (97.7)	
Female	248 (38.9)	1 (0.4)	240 (99.6)	
Smoking index	321.1 ± 537.0	296.6 ± 394.6	321.5 ± 539.2	0.884
Drinking history n(%)	122 (19.2)	3 (2.5)	119 (97.5)	0.413
Hypertension n(%)	171 (26.8)	3 (1.8)	168 (98.2)	0.733
Diabetes mellitus n(%)	73 (11.5)	4 (5.5)	69 (94.5)	0.020
Hyperlipidemia n(%)	43 (67.5)	1 (2.3)	42 (97.7)	0.505
Arrhythmia n(%)	12 (1.9)	0 (0)	12 (100.0)	1
Coronary heart disease n(%)	112 (17.6)	1 (0.9)	111 (99.1)	1
Hypercholesterolemia n(%)	10 (1.6)	0 (0)	10 (100.0)	1
Operative procedure (n%)				<0.001
Left pneumonectomy	23 (3.6)	2 (8.7)	21 (91.3)	
Left upper lobectomy	151 (23.7)	7 (4.6)	144 (95.4)	
Left lower lobectomy	126 (19.8)	0 (0)	126 (100.0)	
Right pneumonectomy	5 (0.8)	0 (0)	5 (100.0)	
Right upper lobectomy	159 (25.0)	1 (0.6)	158 (99.4)	
Right middle lobectomy	30 (4.7)	0 (0)	30 (100.0)	
Right lower lobectomy	117 (18.4)	0 (0)	117 (100.0)	
Right upper and middle	4 (0.6)	0 (0)	4 (100.0)	
lobectomy				
Right middle and lower	22 (3.5)	0 (0)	22 (100.0)	
lobectomy				
Operative approach				0.016
Thoracotomy	154 (24.2)	6 (3.9)	148 (96.1)	
VATS	483 (75.8)	4 (0.8)	479 (99.2)	
Operative time (min)	159.5 ± 58.3	188.7 ± 67.0	159.0 ± 58.1	0.110
Blood loss volume (mL)	182.2 ± 245.6	165.0 ± 127.0	182.5 ± 247.1	0.827
Transfusion n(%)	28 (4.4)	0 (0)	28 (100.0)	1
Infusion volume (mL)	1819.3 ± 726.3	1630.0 ± 459.6	1822.3 ± 729.6	0.409
Duration of drainage (day)	8.3 ± 4.4	10.1 ± 4.3	8.3 ± 4.4	0.184
Hospital stay (day)	21.9 ± 8.6	26.2 ± 5.7	21.9 ± 8.7	0.115
Postoperative complication n(%)	83 (13.0)	1 (1.2)	82 (98.8)	1
Histologic type n(%)				0.719
Small cell carcinoma	15 (2.4)	0 (0)	15 (100.0)	
Adenocarcinoma	385 (60.4)	4 (1.0)	381 (99.0)	
Squamous cell carcinoma	176 (27.6)	5 (2.8)	171 (71.2)	
Adenosquamous carcinoma	3 (0.5)	0 (0)	3 (100.0)	
Bronchiectasis	15 (2.4)	0 (0)	15 (100.0)	
Inflammatory pseudotumor	10 (1.6)	0 (0)	10 (100.0)	
others	33 (5.2)	1 (3.0)	32 (97.0)	
Postoperative LMWH	508 (79.7)	7 (1.4)	501 (98.6)	0.439
anticoagulant therapy n(%)				

Note: Data are mean ± SD, number (%); VATS, video-assisted thoracic surgery; LMWH, low molecular weight heparin.

### Clinical characteristics of postoperative stroke patients

Throughout the study population, the incidence of postoperative stroke was 1.57%. Among the 10 patients with postoperative ischemic stroke, seven had LUL, two had left pneumonectomy, and one had right upper lobectomy (RUL). The patients with postoperative stroke accounted for 5.17% of all patients who underwent LUL and left pneumonectomy. One of these 10 cases had a stroke only after the first postoperative day. Five patients had thrombosis at the LSPV stump as detected by contrast-enhanced CT (CECT). No thrombi were found on CECT of three patients. Two patients refused to undergo chest CECT within 6 months after surgery. Patient details are shown in Table 2.

**Table 2:** Patients with postoperative stroke.

Case	Age (years)/sex	Concomitant conditions	Surgical procedure	Location of the thrombus (contrast enhanced CT)	Operative approach Thoracotomy/VATS	Interval (days)^a^
1	72/M	None	LUL	Not examined	VATS	115
2	59/M	Coronary heart	L pneumonectomy	None	Thoracotomy	28
		disease				
3	58/M	Hypertension	LUL	None	Thoracotomy	129
4	67/M	Diabetes mellitus	L pneumonectomy	LSPV	Thoracotomy	137
5	58/M	Hypertension	LUL	Not examined	VATS	20
6	57/M	Hypertension,	LUL	LSPV	Thoracotomy	122
		Diabetes mellitus				
7	66/F	Diabetes mellitus	RUL	LSPV	VATS	129
8	55/M	Hyperlipidemia	LUL	None	VATS	149
9	73/M	Diabetes mellitus	LUL	LSPV	Thoracotomy	33
10	51/M	None	LUL	LSPV	Thoracotomy	1

M, male; F, female; CT, computed tomography; MRI, magnetic resonance imaging.

L pneumonectomy, left pneumonectomy; LUL, left upper lobectomy.

RUL, right upper lobectomy; LSPV, left superior pulmonary vein.

VATS, video-assisted thoracic surgery.

^a^Time from pulmonary lobectomy to the occurrence of postoperative stroke.

### Predictors of postoperative stroke

The clinical factors of the 10 patients with postoperative stroke were compared with those of the other 627 patients without postoperative stroke. Univariable analysis revealed that LUL and left pneumonectomy (p < 0.001), diabetes mellitus (p = 0.020), and thoracotomy (p = 0.016) were higher in patients with postoperative stroke. Univariable logistic regression analysis demonstrated that LUL and left pneumonectomy (odds ratio, 25.20; 95% CI, 3.17–200.42; P = 0.002) were significant predictors of postoperative stroke. After adjusting for confounders [age, sex, smoking index, drinking history, hypertension, diabetes mellitus, hyperlipidemia, arrhythmia, coronary heart disease, hypercholesterolemia, type of procedure, operative approach (thoracotomy/VATS], LUL, and left pneumonectomy independently predicted stroke (odds ratio, 18.12; 95% CI, 2.12–155.24; P = 0.008). In addition, diabetes mellitus (odds ratio, 6.43; 95% CI, 1.37–30.21; P = 0.019) was also an independent predictor of postoperative stroke (Table 3).

**Table 3:** Multivariable predictors of postoperative stroke.

	Unadjusted		Adjusted	
Variables	OR (95%CI)	P-value	OR (95% CI)	P-value
Age (years)	1.029 (0.96–1.102)	0.423	1.025 (0.942–1.115)	0.569
Sex	5.850 (0.737–46.462)	0.095	4.913 (0.500–48.251)	0.172
Smoking index	1.000 (0.009–1.001)	0.884	0.999 (0.997–1.000	0.147
Drinking history	1.830 (0.466–7,179)	0.386	2.229 (0.417–11.904)	0.348
Hypertension	1.171 (0.299–4.580)	0.821	1.199 (0.240–5.991)	0.825
Diabetes mellitus	5.391 (1.485–19.578)	0.010	6.425 (1.366–30.215)	0.019
Hyperlipidemia	1.548 (0.192–12.507)	0.682	4.567 (0.434–48.122)	0.206
Arrhythmia	0.000 (0.000- —)	0.999	0.000 (0.000- —)	0.999
Coronary heart disease	0.517 (0.065–4.118)	0.533	0.662 (0.067–6.592)	0.725
Hypercholesterolemia	0.000 (0.000- —)	0.999	0.000 (0.000- —)	0.999
Operative approach (Thoracotomy/VATS)	4.855 (1.352–17.435)	0.015	3.872 (0.903–16.607)	0.068
Left upper lobectomy and left pneumonectomy	25.200 (3.168–200.429)	0.002	18.122 (2.116–155.241)	0.008

VATS, video-assisted thoracic surgery; OR odds ratio; CI, confidence interval.

## Discussion

Our study demonstrated that patients who underwent LUL and left pneumonectomy more frequently developed postoperative stroke compared with patients who underwent other operative procedures. In the multivariable-adjusted models, LUL, left pneumonectomy, and diabetes mellitus were independently associated with postoperative stroke. We found that the LSPV thrombosis can be detected in most postoperative stroke patients who underwent LUL and left pneumonectomy, indicating that the LSPV thrombosis may be associated with postoperative stroke.

One retrospective study reported that the LUL procedure was identified as the only risk factor for postoperative stroke^8^, which is partially consistent with our findings. Some reports concluded that thrombosis of the pulmonary vein stump after pulmonary lobectomy may be a new risk factor for embolism^17^. There are two possible explanations for the mechanism of thrombosis. Malm *et al*. reported that the mechanism of thrombosis may be related to endothelial damage during operation and blood stasis caused by blind pulmonary vein stumps^18^. Kwek *et al*. reported that longer pulmonary vein stumps are responsible for thrombosis development^19^. The length of the LSPV is longer than others, and the LSPV has the longest intrapericardial segment^10,20,21^. It was reported that thrombosis in the pulmonary vein stump was detected in 3.3%-3.6% of the patients who underwent lobectomy and in 13.5%-17.9% of those who underwent LUL^9,19^. In our study, among the 8 postoperative stroke patients who underwent CECT after surgery, 5 patients had thrombosis at the LSPV stump, whereas 3 patients had no left upper pulmonary vein thrombosis. Acute renal arterial thrombosis diagnosed 3 days after LUL has been previously reported, which strongly suggests that infarction was associated with surgery, but CECT failed to detect pulmonary vein thrombosis^22^. Therefore, we suspect that the thrombosis moved before the examination and reached the infarct site blocking blood circulation. Even if no thrombus is observed in the pulmonary vein stump, symptomatic embolism within a few days after LUL or left pneumonectomy should still be suspected.

In our research, diabetes mellitus also was a significant predictor of postoperative stroke. Not surprisingly, diabetes mellitus is a well-established independent risk factor for stroke and is associated with poor outcome after stroke, and the mechanism of stroke development secondary to diabetes may be due to cerebrovascular atherosclerosis, cardiac embolism, or rheologic abnormalities^23^. It has been confirmed that diabetes is a risk factor for postoperative stroke in major cardiovascular surgery^24,25^.

Postoperative atrial fibrillation (AF) is generally considered to be associated with postoperative stroke^26^. It was reported that left lobectomy may be a risk factor for AF following pulmonary lobectomy^27^. However, in our study, ten patients with postoperative cerebral infarction did not have a history of AF or postoperative AF. A retrospective study of postoperative stroke and several case reports of postoperative vital organ infarction support our results^8,28–30^. In these reports, LUL was performed leaving the pulmonary vein stump, but no postoperative AF occurred. The other typical stroke risk factors (such as age, smoking, hypertension, hyperlipidemia, and so on) were not found to be predictive of stroke in this study. We consider that it is related to the small number of postoperative stroke patients. Therefore, we are preparing to expand the sample size to confirm the relationship between these risk factors and postoperative stroke in a future study.

The results of our study highlight the importance of taking measures to curtail this risk. If the length of the venous stump is the main cause of thrombosis, the LSPV should be shortened as much as possible to prevent thrombosis. It is reported that the LSPV should be divided in the pericardium to shorten the length of the PV stump^8,9^. However, this method may not be suitable for all patients because it is highly invasive and has technical issues. If the stroke is caused by a thrombosis of the LSPV, we suggest that anticoagulant therapy should be performed before surgery or prolonged postoperative anticoagulant therapy should be considered as thromboembolic prophylaxis to prevent thrombosis especially for patients undergoing LUL or left lung resection. In addition, it is best to perform CECT for routine imaging after surgery. For patients with PV stump thrombosis, anticoagulant therapy should be performed immediately. It is necessary to assess pulmonary vein thrombosis based on the history of surgery for patients with cryptogenic stroke. However, in most instances, the pulmonary vein stump was not checked as a potential origin embolism. Consequently, this relationship might be ignored.

The cause of thrombosis is controversial. Aggressive glucose control through effective drug treatment and modification of other associated risk factors such as blood pressure are critical steps toward effective stroke prevention. In short, the findings might provide useful information for preventing postoperative stroke and guiding clinical decision making in a patient cohort of pulmonary lobectomy surgical patients with postoperative stroke.

Although this is the first relevant study with the largest sample size, there are still several limitations. Firstly, not all patients had routine head MRI or CT examination after surgery; thus, the incidence of asymptomatic postoperative stroke might be underestimated. Secondly, not all patients had postoperative chest CECT. Two patients with postoperative stroke did not undergo postoperative CT examination. We were unable to determine whether the patient had LSPV stump thrombus. Thirdly, the patients who died during the study period were excluded; therefore, we cannot conclude that some deaths were due to postoperative stroke. Finally, the incidence of postoperative stroke is low, and the number of postoperative stroke patients was small in this study. Therefore, a multicenter prospective cohort study with a larger sample size is needed to further determine the relationship between LUL and left pneumonectomy and postoperative cerebral infarction.

## Conclusions

In summary, LUL, left pneumonectomy, and diabetes mellitus are significant risk factors for postoperative stroke. We recommend that further studies should be carried out with respect to the mechanism of postoperative stroke. If it is possible to elucidate this, it is of great significance for the prevention and treatment of postoperative stroke.

## Supplementary information

STROBE Statement—Checklist of items that should be included in reports of cohort studies

## Data Availability

The datasets generated during and/or analysed during the current study are available from the corresponding author on reasonable request.
